# Using a quality of life (QoL)-monitor: preliminary results of a randomized trial in Dutch patients with early breast cancer

**DOI:** 10.1007/s11136-020-02549-8

**Published:** 2020-06-11

**Authors:** R. T. Lugtenberg, M. J. Fischer, F. de Jongh, K. Kobayashi, K. Inoue, A. Matsuda, K. Kubota, N. Weijl, K. Yamaoka, S. R. S. Ramai, J. W. R. Nortier, H. Putter, H. Gelderblom, A. A. Kaptein, J. R. Kroep

**Affiliations:** 1 Department of Medical Oncology, Leiden University Medical Center, Albinusdreef 2, P.O. Box 9600, 2300 RC Leiden, The Netherlands; 2 Department of Medical Psychology, Leiden University Medical Center, Leiden, The Netherlands; 3 Department of Respiratory Medicine, Saitama International Medical Center, Saitama, Japan; 4 Division of Breast Oncology, Saitama Cancer Center, Saitama, Japan; 5 Department of Hygiene and Public Health, Teikyo University Graduate School of Public Health, Tokyo, Japan; 6 Department of Pulmonary Medicine and Oncology, Nippon Medical School, Saitama, Japan; 7 Department of Medical Oncology, HMC Bronovo Hospital, The Hague, The Netherlands; 8 Department of Pulmonology, Leiden University Medical Center, Leiden, The Netherlands; 9 Department of Medical Statistics, Leiden University Medical Center, Leiden, The Netherlands

**Keywords:** Patient-physician communication, Quality of life, Audio-recordings, Breast cancer, Illness perceptions, RCT

## Abstract

**Purpose:**

The diagnosis and treatment of cancer negatively affect patients’ physical, functional and psychological wellbeing. Patients’ needs for care cannot be addressed unless they are recognized by healthcare providers (HCPs). The use of quality of life (QoL) assessments with feedback to HCPs might facilitate the identification and discussion of QoL-topics.

**Methods:**

113 patients with stage I–IIIB breast cancer treated with chemotherapy were included in this randomized controlled trial. Patients were randomly allocated to receive either usual care, or usual care with an intervention consisting of a QoL-monitor assessing QoL, distress and care needs before every chemotherapy cycle visit. Patients completed questionnaires regarding QoL, illness perceptions, self-efficacy, and satisfaction with communication. From the 2nd visit onwards, patients in the intervention arm and their HCPs received a copy of the QoL overview and results were shown in patients’ medical files. Audio-recordings and patients’ self-reports were used to investigate effects on communication, patient management and patient-wellbeing. A composite score for communication was calculated by summing the number of QoL-topics discussed during each consultation.

**Results:**

Use of the QoL-monitor resulted in a higher communication score (0.7 topics increase per visit, *p* = 0.04), especially regarding the disease-specific and psychosocial issues (*p* < 0.01). There were no differences in patient management, QoL, illness perceptions or distress. Patients in the experimental arm (*n* = 60) had higher scores on satisfaction with communication (*p* < 0.05).

**Conclusions:**

Use of a QoL-monitor during chemotherapy in patients with early breast cancer might result in a more frequent discussion of QoL-topics, associated with high levels of patients’ satisfaction.

**Electronic supplementary material:**

The online version of this article (10.1007/s11136-020-02549-8) contains supplementary material, which is available to authorized users.

## Introduction

The diagnosis and treatment of cancer has a severe impact on patients’ quality of life (QoL). Patients typically suffer from physical, functional and psychosocial consequences, some of which may persist after completion of treatment. Although it is widely acknowledged that QoL is affected by disease and treatment, the individual patients’ needs cannot be adequately addressed unless these problems are recognized by their healthcare providers (HCPs). Research has demonstrated that problems and needs of patients with cancer are not always adequately identified [1–4]. Nowadays, the impact of cancer diagnosis and treatment on patients’ QoL is regularly addressed by HCPs and patients during consultations. However, the emphasis is often on treatment and symptoms [5, 6]. Previous communication studies suggested that the majority of patients with cancer wish to discuss psychosocial matters during the consultation, whereas a minority (25–35%) of patients would like to discuss these issues only if their HCP initiates the discussion [7, 8]. However, in daily practice, only a limited amount of discussion time is dedicated to psychosocial matters leaving little time for patients to express their feelings to their HCPs or receiving the emotional support they need [9–11]. At the same time patients are more willing to discuss their health concerns, particularly sensitive issues, when they have a longer relationship with their HCP [12, 13].

Characteristics and perceptions of patients and HCPs, such as age, assertiveness, illness perceptions, medication beliefs, or health literacy, can influence the communication. Patients’ self-efficacy in patient-physician interactions plays an important role in question asking during the consultation, active participation in medical decision making and knowledge about their disease [14, 15]. The use of patient-reported outcomes (PRO) questionnaires could help patients to increase self-efficacy and overcome barriers to participate effectively during consultations. Furthermore, higher self-efficacy for coping with symptoms was associated with greater functional, emotional and social wellbeing in a study among patients with breast cancer taking adjuvant endocrine therapy [16].

It has been suggested that the use of validated QoL assessments in daily practice is likely to facilitate the detection and discussion of otherwise unidentified issues [17–20]. This will lead to improved communication between patients and HCPs, a necessary prerequisite to reach better QoL and improved satisfaction regarding communication with their HCPs [17].

The department of Medical Oncology of Leiden University Medical Center, The Netherlands in collaboration with the departments of Breast Oncology and Respiratory Medicine of the Saitama Cancer Center, Saitama, Japan has developed a QoL-monitor specifically for patients with breast cancer [21]. This monitor consists of a general and breast cancer specific questionnaire that assesses QoL, distress and care needs during and after treatment. We have shown in previous studies that patients and HCPs generally had a positive attitude towards using the monitor [22]. The purpose of this randomized study was to investigate the beneficial effects of the QoL-monitor on aspects of communication, medical care and patient outcomes in patients with early breast cancer in the Netherlands and Japan who were treated with adjuvant or neo-adjuvant chemotherapy. The primary objective of this study was to assess the extent to which QoL-topics were discussed. Secondary objectives included the effects on patient management; length of consultations; patients’ perceived efficacy to communicate with HCPs; patients’ satisfaction regarding communication with their HCP; QoL and distress; and finally patients’ perceptions of their illness. In this report we present the results of the patients treated in the Netherlands.

## Methods

### Study design

**Fig. 1:**
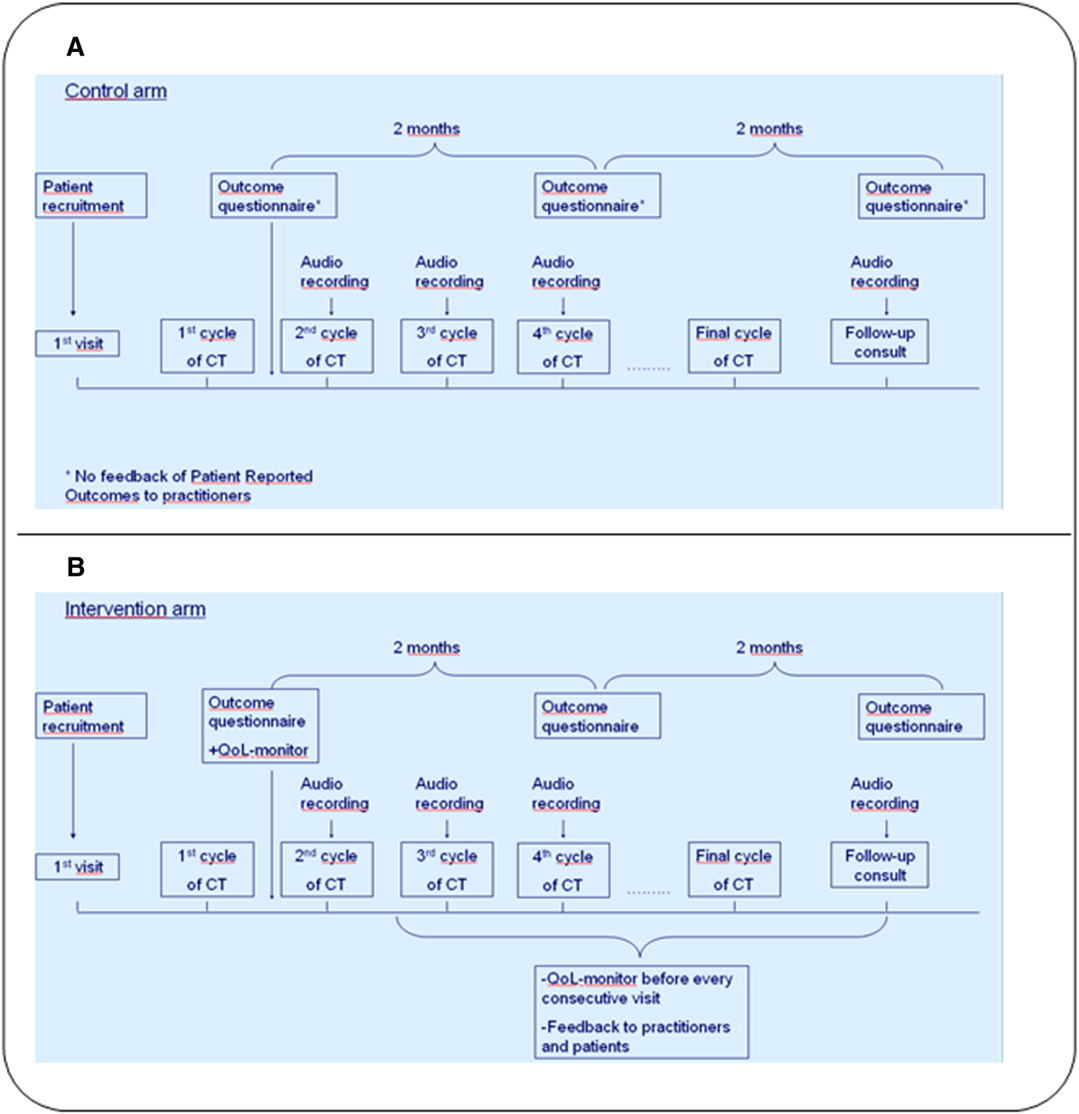
Timeline of assessments for patients in the control arm (**a**) and the experimental condition (**b**). The outcome questionnaire included assessment of QoL, illness perceptions, self-efficacy, satisfaction with communication and distress. The QoL-monitor consists of general and breast cancer specific QoL questionnaires, distress and care needs during and after treatment. *CT* chemotherapy

**Fig. 2:**
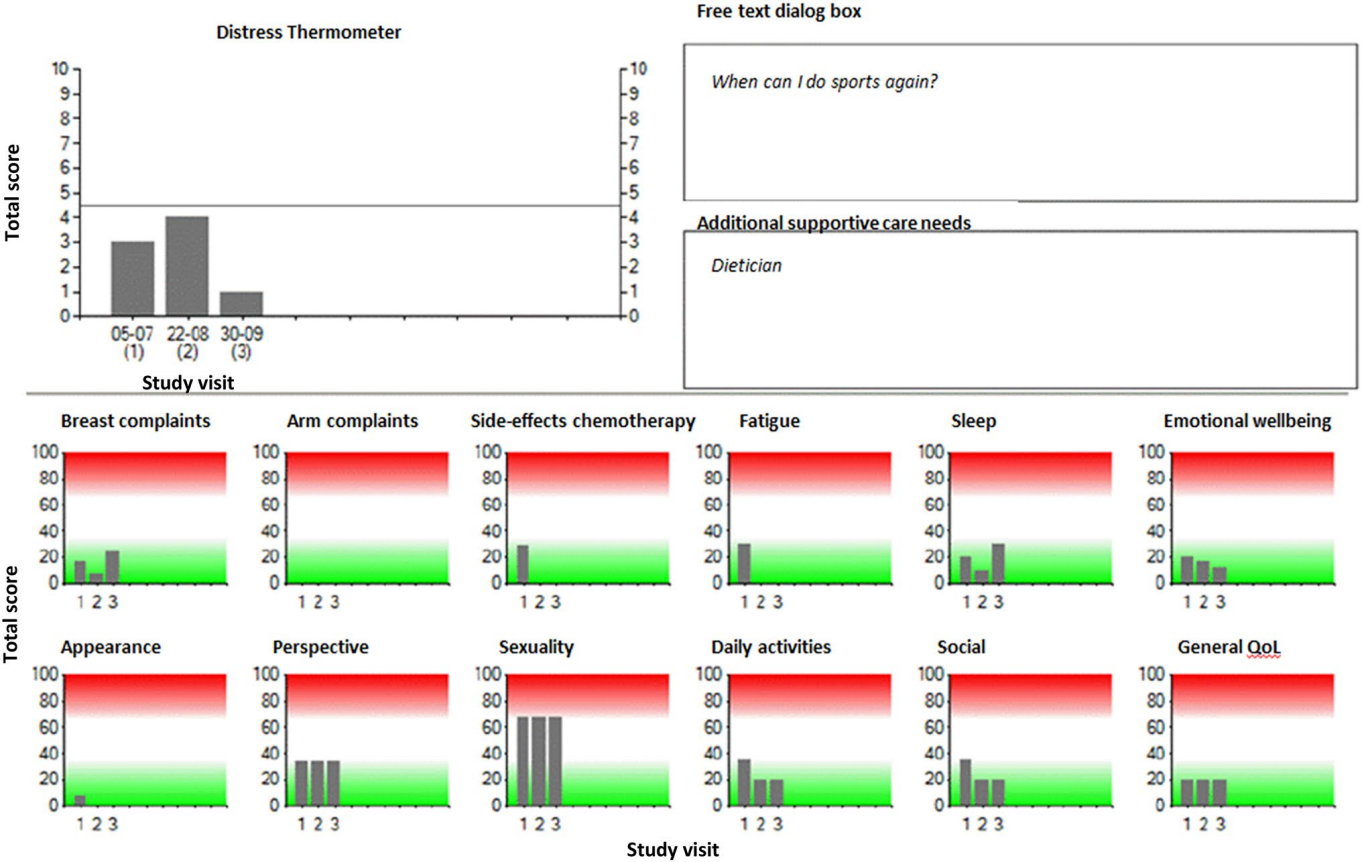
Graphic overview of the summarized QoL-monitor results. The summarized QoL-monitor included the results of the Care Notebook, EORTC BR-23 questionnaire, distress thermometer, free text dialog box and additional supportive care needs and were stored in the medical files

Patients with early breast cancer eligible for neo-adjuvant of adjuvant chemotherapy were asked to participate in this randomized trial. Patients were randomized to the intervention or to standard care with a ratio of 1:1. To secure this ratio, a permuted block randomization was used, with a block size of four. Stratification was performed for type of chemotherapy (adjuvant or neo-adjuvant treatment) and for treatment site. In the intervention arm patients received usual care combined with the QoL-monitor. This consisted of a standardized questionnaire assessing QoL, distress and care needs before every consecutive hospital visit for chemotherapy as is depicted in Fig. 1. An overview of the monitor results were fed back to the HCP’s and patient and implemented in the digital medical files as is depicted in Fig. 2. In this graphic overview, results were shown for each of the items of the monitor. This study was conducted as part of a larger trans-cultural study in the Netherlands and Japan.

### Patients

Patients were recruited from the Leiden University Medical Center, Leiden, and the Hague Medical Center (HMC), location Bronovo, during their first visit with their HCP before the start of chemotherapy. Eligible patients willing to participate in this study gave informed consent. Eligibility criteria for patients with breast cancer were: invasive ductal or lobular carcinoma stage I–III; performance status 0–1; and scheduled to receive neo-adjuvant or adjuvant chemotherapy. Exclusion criteria were: distant metastases and receiving hormonal therapy only.

### Healthcare providers

Before the start of the study, a plenary session was organized to inform physicians and nurse practitioners about the aim and design of the study, to reinforce knowledge about the interpretation of QoL scores, and to discuss suggestions for how to use the monitor in daily practice. HCPs could find the monitor results in the digital medical file; they received a message 1 day before the medical visit to inform them that the patients file contained updated information with regard to their QoL results.

### Data collection

At four hospital visits [before 2nd (study visit 1), 3rd (study visit 2) and 4th (study visit 3) chemotherapy cycle and at the first follow-up visit after chemotherapy (study visit 4)] audio-recordings were used to investigate the benefits of using the QoL-monitor for the communication and patient management, and to examine its effect on the length of visit (see Fig. 1). All visits were regular consultations with their HCP as part of standard care during breast cancer treatment with chemotherapy. The first recording was served as a baseline assessment in the analyses to determine possible baseline differences in both study arms. The content of the audio-recordings was independently analyzed by one observer (RL) who was blinded to patient identity and group assignment. To establish interrater reliability a random sample of 40% of the audiotapes was analyzed by a second observer (FdJ). Breast cancer specific checklists were used to investigate the number of QoL-topics discussed during the consultation (Supplementary Material). The checklist consisted of nineteen questions that refer to a specific symptom or domain of functioning. These items are derived from the function and symptoms scales of the cancer generic EORTC-QLQ C30 and breast cancer specific EORTC BR-23 QoL questionnaires. The checklist included the following topics: pain, fatigue, dyspnea, nausea, insomnia, appetite, constipation, diarrhea, general side-effects of chemotherapy, complaints of the arm or breast, physical functioning, role functioning, cognitive functioning, emotional functioning, social functioning, sexuality, body image and future perspective [23]. A dichotomous (yes/no) scoring system was used to indicate whether the topic was discussed. A composite score for communication was calculated by summing the number of QoL-topics discussed during each consultation. In addition, per topic it was determined who initiated the discussion of the topic, and which actions were taken. To establish the level of agreement between observers and patients in our study, patients filled out the same checklist after the third study visit. Patients received the checklist with a pre-paid return envelope immediately after the consultation. Patients were asked to complete it at home and return it to the researchers as soon as possible. To investigate the secondary research questions all participants completed an outcome questionnaire at three moments. The first questionnaire was completed before the start of the second chemotherapy, the second questionnaire 2 months later and the third questionnaire 4 months later. Sociodemographic and clinical characteristics were obtained from patients’ medical records.

### QoL-monitor (intervention arm)

The instrument included the following items:

1. The European Organization for Research and Treatment of Cancer BR-23 breast cancer questionnaire [24]. This 23-item questionnaire covers both physical and psychosocial domains and is widely used to assess breast cancer-related problems. Symptom scales (arm, breast, upset by hair loss and side-effects of systemic therapy) and functional scales (body image, sexual functioning and future perspective) were collected and scored on 4-point Likert scales. Domains are calculated by transforming single or combined questions into a 0–100 scale.
2. The Care Notebook (CNB) [21]. This 24-item instrument was designed and validated for measuring patients’ QoL in the daily oncology practice. The questionnaire contains items on symptoms and physical conditions, emotional status, and items related to functioning and life situations (two items each on daily physical functioning and social functioning, and four items about subjective QoL). An 11-point (0–10) scale is used for each question.
3. The National Comprehensive Cancer Network (NCCN) Distress Thermometer (DT) [25]. The DT is a single-item instrument that relates to the level of distress (range 0–10) a patient has experienced in the past week. A validation study of the DT in a Dutch sample of cancer patients showed that a score of 5 or higher may be regarded as a sign for elevated distress [26].
4. One free text dialog box. Here, patients were invited to list topics or specific questions they would like to discuss with their HCP during their next hospital visit.
5. One question assessing additional supportive care needs. Patients could indicate whether they would like to discuss specific complaints or their condition in general with persons other than their primary care provider. A list of persons was provided.

At the start of the study patients were asked if they wanted to complete the QoL-monitor online or printed on paper. If they chose the paper version the questionnaires were sent by post, about 7 days before the next consultation with their HCP. Patients who chose to complete the questionnaire online received an email with a link that led them to the monitor, a few days before the next consultation with their HCP. All patients were instructed to answer the questions by checking the box next to the answer that best reflected their opinion.

Answers were summarized and processed into an overview, including results from the previous completed monitors. Starting from the second study visit (before the third cycle of chemotherapy) results of the QoL-monitor were stored in the medical files.

### Outcome questionnaire to investigate secondary objectives (all patients)

The questionnaire included assessment of the following items:

1. *Quality of life* assessed with the European Organization for Research and Treatment of Cancer-Core Quality of Life Questionnaire (EORTC-QLQ C30) [27]. This 30-item test comprises five function scales (physical, emotional, cognitive, social, and role), one global health related QoL scale, three symptom scales and six single items. All scores are transformed to a 0–100 scale.
2. *Illness perceptions* assessed with the Brief Illness Perception Questionnaire (BIPQ) [28]. This validated questionnaire consists of eight single-item domains that relate to patients’ cognitive (e.g., consequences, perceived controllability) and emotional representations of the illness. All domains are scored on a 0–10 scale.
3. *Self-efficacy* assessed with the Perceived Efficacy in Patient-Physician Interactions (PEPPI) questionnaire [29]. Questions on this 10-item scale are summed (range 10–50). Higher scores indicate greater perceived personal efficacy in interacting with HCP’s.
4. *Satisfaction with communication* assessed with the Medical Care Questionnaire-Communication (MCQ-C) subscale [30]. The test consists of five items answered on a 5-point scale. Scores are transformed to a 0–100 scale, with higher scores indicating greater satisfaction.
5. *Distress* assessed with the Distress Thermometer (DT) and the Hospital Anxiety and Depression scale (HADS) [25, 31]. The DT is a single-item instrument that relates to the level of distress (range 0–10) a patient has experienced in the past week. The HADS is a 14-item questionnaire with seven questions pertaining to anxiety and seven items that assess symptoms of depression. Questions are answered on a 4-point scale and summed for each subscale (range 0–21).

### Evaluation of the QoL-monitor (intervention arm)

The evaluation consisted of seven questions. Patients were asked if the monitor was burdensome or difficult to complete (both scored on a 1–5 Likert scale, in which higher scores indicate worse usefulness). Furthermore patients were asked to indicate to what degree they perceived the monitor was useful for the interaction with their HCPs and if the monitor was suitable for documentation (both scored on a 1–5 Likert scale, in which higher scores indicate better usefulness). In addition, patients were asked to indicate the average time it took them to complete the monitor, whether patients thought the monitor should be introduced as a standard instrument during treatment and if they had suggestions to improve the monitor.

### Statistical methods

The hypothesis was that the use of the QoL-monitor resulted in more QoL-related topics being discussed during the consultations with HCPs. The study was powered to detect a difference of 0.8 items, based on results from the previous study of Detmar et al. [7]. With a power of 80% and α of 0.05, a minimum of 110 participants were needed per study arm. Anticipating a 20% drop-out rate, we aimed to include a total of 280 patients in both Japan and the Netherlands.

Descriptive analyses were used to summarize patients’ sociodemographic and medical characteristics. Parametric and nonparametric tests were used to compare the intervention and control groups at baseline. The level or agreement between the two observers was assessed by percentage of agreement and with Cohen’s κ.

Linear mixed models were used to investigate longitudinal changes in primary and secondary outcomes between both study arms. The models included fixed effects for study arm, baseline measurements at the first study visit, time (consecutive study visits or questionnaires), and the interaction for time*study arm, and a random intercept for patients. Possible covariates (age, tumor stage, hormonal status, HER2 status, neo-adjuvant vs. adjuvant chemotherapy, previous radiotherapy, partner relation and employment were identified by univariate regression analyses. Covariates were entered in the model as a fixed effect when showing a univariate relationship (*p* < 0.1) with the outcome measure.

Post-hoc analyses were done to explore whether the use of the monitor increased the probability of a particular QoL domain being discussed multivariate logistic regression was used. The 19-items of the checklist were subdivided in four QoL domains as follows:

chemotherapy side-effects: pain, fatigue, dyspnea, nausea, sleep, appetite, constipation, diarrhea and general side-effects of chemotherapy; Loco-regional symptoms of breast cancer: complaints of the arm + complaints of the breast; Functional limitations: physical functioning, role functioning; Psychosocial functioning: cognitive functioning, emotional functioning, social functioning, sexuality, body image and future perspective. The same covariates as in the mixed model were tested for inclusion in univariate logistic regression models for each of the four QoL domains with a threshold of *p* < 0.1. The significance level was set at *p* < 0.01 for the multivariate analysis, to adjust for multiple testing. All analyses were performed using SPSS version 23.0 (IBM, Armonk, NY).

## Results

### Patients

Between July 2012 and May 2016, 116 patients provided informed consent. Three patients were ineligible, resulting in randomization of a total of 113 patients (Fig. 2). Patients’ sociodemographic and clinical characteristics are summarized in Table 1. The median age was 51 years (range 22–72). Most patients were treated for invasive ductal (85%), hormone-receptor positive (74%) and stage II (67%) breast cancer. There were no significant differences between patients in the control versus the intervention group.

**Table 1:** Sociodemographic and clinical characteristics

Characteristic	Intervention (*n* = 60)	Control (*n* = 53)	*N*
Age (years)^a^	51.7 (10.9)	52.1 (9.6)	113
Partnered/married^b^	41 (77.4%)	49 (81.7%)	113
Children^b^			113
No	10 (16.7%)	11 (20.8%)	
Yes	47 (78.3%)	40 (75.5%)	
Unknown	3 (5.0%)	2 (3.8%)	
Employed at time of diagnosis^b^	42 (75.0%)	34 (73.9%)	102
Cancer subtype^b^			112
Invasive ductal	50 (83.3%)	45 (86.5%)	
Invasive lobular	5 (8.3%)	6 (11.5%)	
Other	5 (8.3%)	1 (1.9%)	
Cancer stage^b^			110
I	12 (20.3%)	10 (19.6%)	
II	39 (66.1%)	35 (68.6%)	
III	8 (13.6%)	6 (11.8%)	
ER and/or PR positive^b^	46 (76.7%)	37 (69.8%)	113
HER2 positive^b^	8 (13.3%)	15 (28.3%)	113
Triple-negative breast cancer^b^	12 (20.0%)	8 (15.1%)	113
Timing of chemotherapy^b^			113
Adjuvant	30 (50.0%)	25 (47.2%)	
Neo-adjuvant	30 (50.0%)	28 (52.8%)	
First cycle chemotherapy^b^			113
TAC	24 (40.0%)	24 (45.3%)	
AC	22 (36.7%)	19 (35.8%)	
FEC	12 (20.0%)	6 (11.3%)	
TC	2 (3.3%)	2 (3.8%)	
PTCptz	–	2 (3.8%)	
Previous radiotherapy^b^	15 (25.0%)	15 (28.3%)	113

*ER* estrogen receptor, *PR* progesterone receptor, *HER2* human epidermal growth factor receptor 2, *TAC* docetaxel, adriamycin and cyclophosphamide, *AC* adriamycin and cyclophosphamide, *FEC* fluorouracil, epirubicin and cyclophosphamide, *TC* docetaxel and cyclophosphamide, *PTCptz paclitaxel, trastuzumab, carboplatin and pertuzumab*

^a^Means (SD)

^b^Frequencies (%), some percentages may not total 100 because of rounding

### Level of agreement

The content of 144 audio fragments from 43 different patients were independently analyzed by both observers (Table 2). Observer 1 (RL) is a medical doctor and observer 2 (FdJ) is a master student in health psychology. A high level of agreement was reached on all the HRQL-items being discussed, with an average percentage of agreement of 95% (range 88–100) and a Cohen’s κ of 0.89 (range 0.65–1.00). At the third study visit, the level of agreement between patients and observers could be analyzed in 62 patients (Table 3). Moderate to excellent levels of agreement were seen between observers and patients. Ranging from 60 to 65% for topics like fatigue, pain and physical activities to 94–97% for the topics regarding physical appearance and sexuality. The Cohen’s κ values varied between 0.12 (breast) to 0.72 (dyspnea), indicating a poor to good range of agreement. Low values of Cohen’s κ were particularly seen in QoL-items that were discussed in almost every consultation, or in items only hardly discussed.

**Table 2:** Agreement between observer ratings of audio-recordings from 144 audio fragments of 43 patients

HRQL topic	Frequency discussed during 1 or more study visits according to rater 1 (%)	Frequency discussed during 1 or more study visits according to rater 2 (%)	Cohen’s κ	Agreement (%)
Pain	40	38	0.81	91
Fatigue	50	49	0.89	94
Dyspnea	15	15	0.97	99
Nausea	65	65	0.97	99
Sleep	23	23	0.92	97
Appetite	35	33	0.86	94
Constipation	39	36	0.91	96
Diarrhea	15	14	0.94	99
Side-effects	97	96	0.79	99
Arm	19	19	0.84	95
Breast	20	19	0.85	95
Physical activities	37	39	0.84	92
Daily activities	33	37	0.80	91
Social	10	10	0.85	97
Concentration	6	6	0.94	99
Emotions	34	40	0.84	92
Sexuality	4	4	1.00	100
Appearance	6	7	0.65	96
Perspective	41	42	0.74	88
Mean			0.89	95

**Table 3:** Patient versus observer ratings of HRQL-related topics discussed during 3rd study visit (*n* = 62)

HRQL topic	Frequency discussed according patient (%)	Frequency discussed according to rater 1 (%)	Frequency discussed according to rater 2(%)	Cohen’s κ Pt-Obs1	Cohen’s κ Pt-Obs2	Cohen’s κ Obs1–Obs2	Agreement (%) Pt-Obs1	Agreement (%) Pt-Obs2	Agreement (%) Obs1–Obs2
Pain	44	34	31	0.26	0.39	0.78	65	71	90
Fatigue	66	45	44	0.34	0.32	0.90	66	65	95
Dyspnea	24	27	21	0.66	0.72	0.83	87	90	94
Nausea	63	66	53	0.37	0.41	0.74	71	71	87
Sleep	42	37	37	0.43	0.50	0.93	73	76	97
Appetite	45	29	29	0.33	0.46	0.77	68	74	90
Constipation	44	42	42	0.57	0.51	0.93	79	76	97
Diarrhea	23	15	10	0.53	0.42	0.77	86	84	95
Side-effects	74	97	98	− 0.06^a^	0.09^a^	− 0.03^a^	71	76	95
Arm	36	32	32	0.64	0.71	0.85	84	87	94
Breast	19	16	13	0.12	0.17	0.74	74	77	94
Physical activities	19	36	44	0.22	0.12	0.63	68	60	82
Daily activities	31	36	39	0.38	0.40	0.79	73	73	90
Social	18	10	10	0.53	0.53	0.82	89	89	97
Concentration	10	2	3	− 0.03^a^	− 0.05^a^	0.66^a^	89	87	98
Emotions	28	24	29	0.32	0.40	0.79	74	75	92
Sexuality	7	3	3	0.65^a^	0.65^a^	1.00^a^	97	97	100
Appearance	5	2	2	− 0.03^a^	− 0.03^a^	1.00^a^	94	94	100
Perspective	21	21	37	0.51	0.39	0.62	84	74	84
Mean				0.50	0.51	0.84	78	79	93

^a^Cohen’s κ not reliable because of very low or high prevalence

### Missing data

**Fig. 3:**
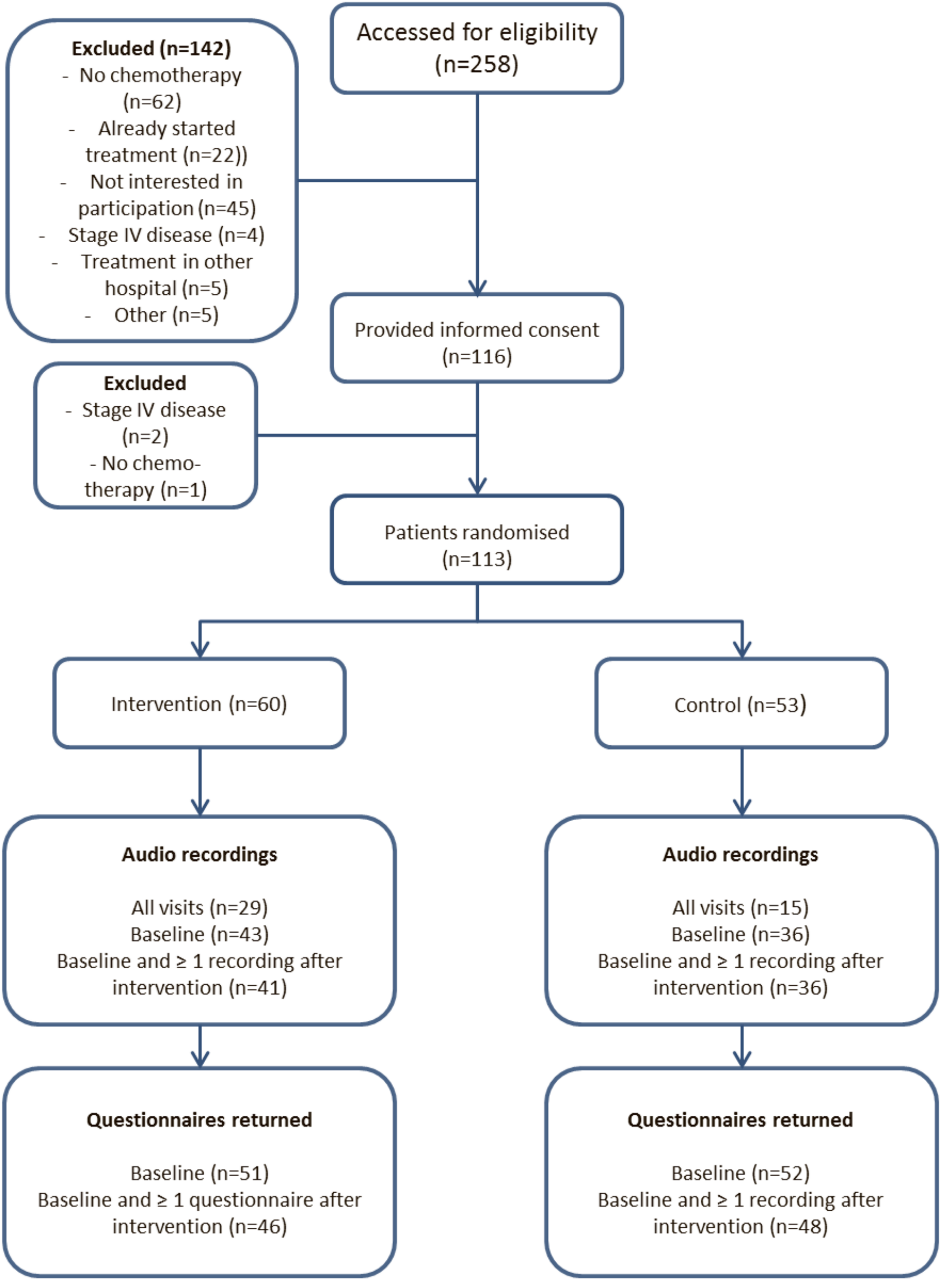
Flowchart

As a consequence of missing audio-recordings, 77 patients (68%) were available for investigating effects of the intervention on communication, patients’ management and length of study visits (Fig. 3). Evaluable patients had an audiotaped first study visit (before the 2nd chemotherapy) and at least one or more recording in the consecutive study visits. 94 out of 113 patients (83%) returned their outcome questionnaires at baseline and at least one time at the other two time points and were eligible for analysis.

#### Primary outcome

**Fig. 4:**
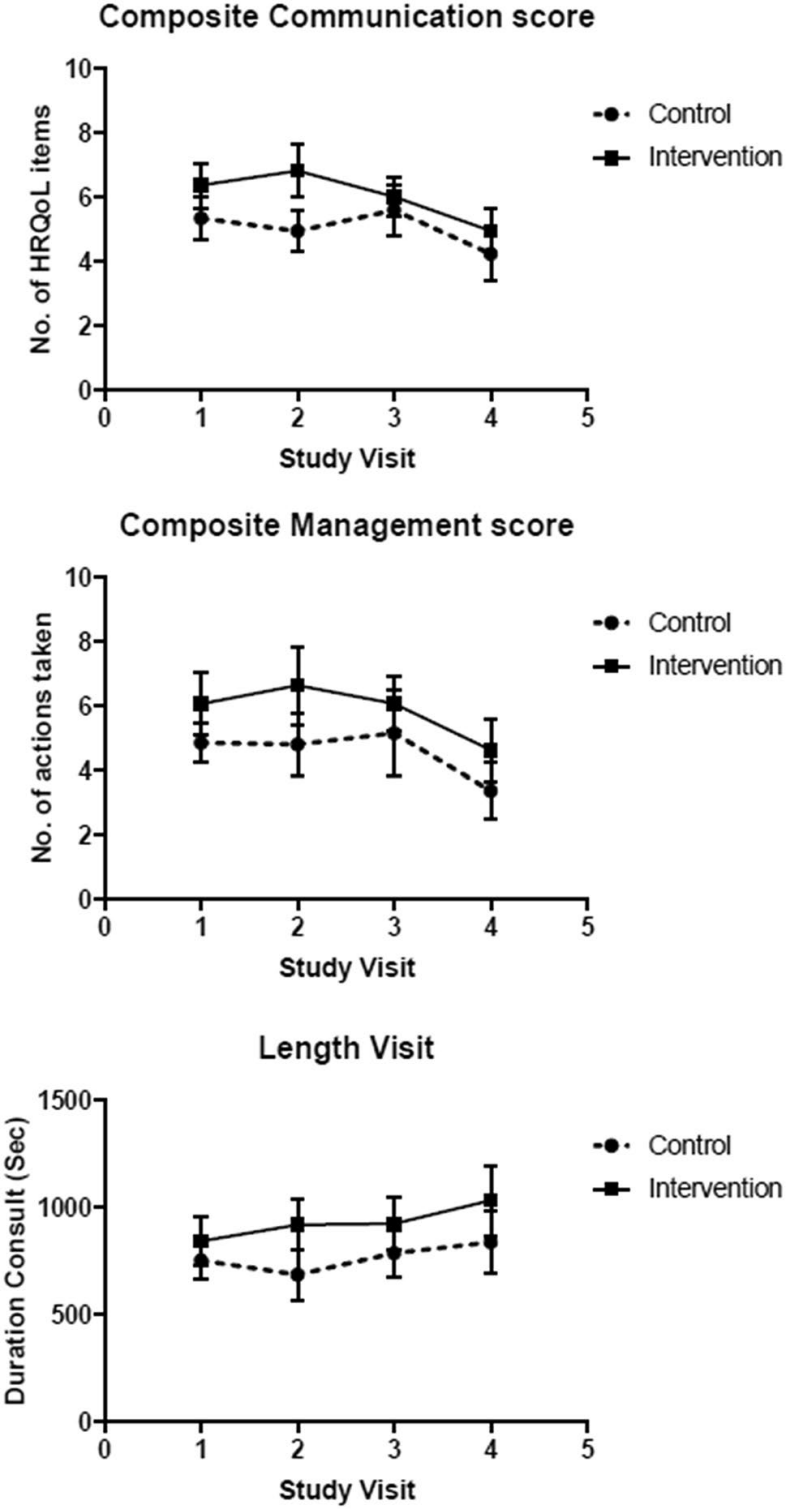
Change in number of QoL-topics discussed (communication score), number of actions taken (management score) and length of study visits

At the first study visit (baseline measure) the mean (SD) composite communication score was 5.36 (1.9) in the control group and 6.39 (2.2) in the intervention group. The composite communication score was 0.7 points higher on average, in the intervention group at the second, third and final study visit (*p* = 0.039, Table 4), with a range of scores between 1 and 13. With 19 different topics on the checklist the theoretical range was 0–19. Figure 4 illustrates the effect of the intervention after introducing the feedback of the QoL overview at study visit 2. The QoL-topics were categorized in four domains: side-effects of chemotherapy, disease-specific complaints (e.g., breast and arm), functional (e.g., activities) and psychosocial issues. The use of the QoL-monitor increased the probability of the disease-specific (*p* ≤ 0.01) and psychosocial (*p* ≤ 0.01) items being discussed (Table 5).

**Table 4:** Mean scores on communication, management and visit length per group and results of linear mixed-model analysis

	Study visit 1 (baseline measure) Mean (SD)	Study visit 2 Mean (SD)		Study visit 3 Mean (SD)	Study visit 4 Mean (SD)	Mean difference per visit (95% CI)	Linear mixed-model analysis (*p*-value)
Composite communication score (no. of HRQL topics discussed)							
Intervention	6.39 (2.2)	6.84 (2.4)		6.03 (1.9)	4.97 (1.9)	0.69 (0.04–1.35)	0.039
Control	5.36 (1.9)	4.96 (1.6)		5.61 (2.2)	4.25 (2.0)		
Composite management score (no. of taken actions)							
Intervention	6.07 (3.0)	6.65 (3.7)		6.08 (2.7)	4.64 (2.77)	0.87 (− 0.13 to 1.87)	0.087
Control	4.86 (1.8)	4.81 (2.5)		5.16 (3.6)	3.38 (2.12)		
Length visit (s)							
Intervention	843 (374)	920 (351)		924 (379)	1033 (459)	146 (46- 246)	0.005
Control	754 (268)	687 (301)		787 (294)	838 (338)

**Table 5:** Logistic regression analysis of separate QoL domains. Increased probability of the topic being discussed when using the QoL-monitor (total group) during study visit 2, 3 or 4

	Total			Study visit 2			Study visit 3			Study visit 4		
	Control *n* (%)	Intervention *n* (%)	*p*-value	Control *n* (%)	Intervention *n* (%)	*p*-value	Control *n* (%)	Intervention *n* (%)	*p*-value	Control *n* (%)	Intervention *n* (%)	*p*-value
Side-effects chemo	36 (100)	41 (100)	n/a	27 (100)	37 (100)	n/a	31 (100)	39 (100)	n/a	22 (92)	32 (97)	0.40^f^
Loco-regional breast	18 (50)	32 (78)	**< 0.01**^a^	8 (30)	11 (30)	0.73^b^	11 (36)	18 (46)	0.41^d^	6 (25)	19 (58)	** < 0.05**^e^
Functional	25 (69)	34 (83)	0.17^f^	11 (41)	23 (65)	0.09^f^	16 (52)	22 (56)	0.69^f^	14 (58)	17 (52)	0.41^e^
Psycho-logical /social	23 (64)	37 (90)	** < 0.01**^f^	12 (44)	24 (65)	0.08^c^	13 (42)	24 (62)	0.11^f^	14 (58)	24 (73)	0.26^f^

Covariates tested for univariate relationship (*p* < 0.1) are: age, tumor stage, hormonal status, HER2 status, neo-adjuvant vs. adjuvant chemotherapy, previous radiotherapy, partner relation, employed included covariates

Bold indicates the *p*-value is < 0.05, there is a statistically significant increased probability of the topic being discussed when using the QoL-monitor

^a^Tumor stage, neo-adjuvant vs adjuvant, radiotherapy

^b^Tumor stage, neo-adjuvant vs adjuvant

^c^Tumor stage, neo-adjuvant vs adjuvant, radiotherapy, partner relation

^d^Neo-adjuvant vs adjuvant

^e^HER2 status

^f^No relevant covariates

### Secondary study measures

#### Patient management

The mean number of actions taken at each study visit (the composite management score) did not differ between study arms (Table 4). Actions that could be taken were for example: counseling, referrals, prescription of medication or advice. As with the composite communication score, the number of taken actions decreased in time in all patients from the third visit onwards (Fig. 4). Between-group differences were not seen in the type of actions taken. Most patients received counseling or advice. Prescriptions of medication, referrals or additional medical tests were observed less frequent.

#### Length of study visits

Patients in the intervention group had longer duration of consultations at the second, third and final study visit, with an average difference of 2 min and 26 s (*p* = 0.005, Table 4). Whereas the number of QoL-topics discussed and the number of actions taken decreased over time, the length of the study visits increased during the consecutive study visits (Fig. 4).

### Outcome questionnaire

All these measures showed no effect of the intervention, no differences over time and the baseline measure as the most important predictor for the outcome measure.

#### Quality of life, illness perceptions and emotional distress

There were no differences between study groups on any of the EORTC-QLQ C30 scales or items, on the BIPQ, on the Distress thermometer and HADS questionnaires. Baseline scores did not differ among study groups. Also, no time effect was observed (Table 6).

**Table 6:** Quality of Life, EORTC-QLQ C30, Brief Illness Perception Questionnaire (BIPQ), Distress thermometer and HADS, Self-efficacy, Perceived Efficacy in Patient-Physician Interactions (PEPPI), The Medical Care Questionnaire-Communication subscale (MCQ-C), mean scores (SD)

	Baseline		During treatment		Follow-up		Between group differences*
	Intervention	Control	Intervention	Control	Intervention	Control	
QoL							
Functional measures							
Physical	85.9 (12.6)	85.7 (14.6)	77.4 (16.4)	81.1 (16.3)	76.6 (16.4)	79.1 (16.0)	n.s
Role	66.7 (28.1)	68.6 (29.8)	58.5 (25.3)	64.4 (30.9)	57.2 (26.3)	66.3 (25.6)	n.s
Emotional	79.6 (17.9)	74.4 (17.6)	74.2 (19.8)	75.7 (19.0)	75.2 (20.8)	76.0 (22.6)	n.s
Social	74.8 (22.9)	76.9 (25.0)	72.1 (22.8)	71.1 (27.6)	73.5 (20.1)	72.9 (19.9)	n.s
Cognitive	78.3 (23.1)	77.0 (22.1)	76.5 (21.5)	76.3 (23.4)	72.7 (21.0)	72.1 (22.6)	n.s
Global health	69.7 (17.4)	68.6 (21.7)	62.7 (19.8)	60.7 (21.6)	61.9 (19.0)	63.6 (17.5)	n.s
Symptoms							
Fatigue	40.7 (24.1)	40.0 (24.2)	47.2 (21.5)	44.7 (24.9)	43.2 (22.6)	40.8 (26.8)	n.s
Pain	16.3 (20.7)	16.3 (24.4)	23.6 (31.8)	17.4 (24.1)	25.0 (28.4)	22.5 (20.9)	n.s
Nausea	15.7 (26.5)	15.4 (23.8)	9.8 (18.2)	10.7 (14.7)	6.8 (15.4)	3.5 (7.8)	n.s
Appetite loss	20.9 (27.5)	23.7 (30.5)	17.9 (27.0)	24.2 (23.1)	9.1 (18.1)	11.6 (17.6)	n.s.
Constipation	29.4 (33.8)	19.9 (29.7)	19.5 (27.9)	17.8 (22.0)	15.9 (26.4)	14.7 (22.2)	n.s
Diarrhea	15.0 (32.2)	23.1 (32.7)	17.9 (24.8)	8.3 (21.7)	10.6 (21.3)	13.2 (27.4)	n.s
Insomnia	34.0 (32.3)	32.7 (31.3)	36.6 (33.2)	31.1 (31.7)	31.8 (24.9)	34.9 (34.8)	n.s
Dyspnea	14.4 (23.3)	12.8 (20.0)	30.0 (28.0)	23.0 (23.4)	24.2 (31.6)	24.8 (25.3)	n.s
Financial impact	11.1 (25.5)	15.4 (27.6)	10.0 (20.3)	13.3 (24.0)	13.6 (24.2)	15.5 (30.3)	n.s
BIPQ							
Consequences	7.4 (1.9)	6.7 (2.2)	6.8 (2.2)	6.7 (2.5)	6.5 (2.3)	6.7 (2.1)	n.s
Timeline	5.4 (2.8)	5.9 (3.2)	5.5 (2.8)	5.7 (2.6)	6.1 (3.1)	5.6 (3.0)	n.s
Personal control	4.7 (3.1)	4.7 (2.6)	4.6 (2.7)	3.8 (2.9)	4.7 (2.9)	4.0 (2.6)	n.s
Treatment control	8.8 (1.0)	8.5 (1.5)	8.6 (1.4)	8.0 (1.8)	8.4 (1.7)	8.3 (1.5)	n.s
Identity	4.2 (2.2)	3.3 (2.2)	5.2 (2.2)	4.1 (2.6)	5.4 (2.2)	5.2 (2.4)	n.s
Concern	5.5 (2.6)	6.2 (2.7)	5.0 (2.3)	5.8 (2.7)	5.3 (2.4)	6.0 (2.5)	n.s
Understanding	6.8 (2.3)	6.8 (2.6)	6.8 (2.0)	6.9 (2.0)	7.0 (2.4)	6.8 (2.2)	n.s
Emotional response	4.8 (2.5)	5.2 (2.5)	4.3 (2.5)	5.3 (2.7)	5.3 (2.3)	5.5 (2.5)	n.s
Distress thermometer							
HADS	4.3 (2.2)	3.7 (2.3)	4.9 (2.1)	4.0 (2.5)	4.2 (2.0)	4.7 (2.3)	n. s
Anxiety	10.4 (1.4)	9.9 (1.8)	10.6 (2.0)	10.2 (1.5)	10.4 (1.5)	10.1 (1.9)	n.s
Depression	10.8 (1.3)	10.7 (1.9)	10.5 (1.8)	10.6 (1.5)	10.6 (1.5)	10.8 (1.5)	n.s
PEPPI	44.4 (5.2)	42.5 (5.8)	42.8 (9.7)	43.8 (6.7)	45.5 (4.3)	43.6 (5.5)	n.s
MCQ-C	70.1 (21.2)	64.0 (18.6)	72.6 (18.4)	64.1 (20.3)	74.8 (18.6)	65.0 (18.3)	n.s

*Mixed model analysis

#### Self-efficacy

High levels of self-efficacy were seen for all participating patients, with high PEPPI-scores at baseline, during treatment and at follow-up (mean scores ranging from 42.5 to 45.5, Table 6). The PEPPI-score at baseline was a strong contributor in the mixed model for the consecutive PEPPI-scores (*p* < 0.001). Also patients’ age was a significant covariate, with higher scores in younger patients (*p* < 0.05). No correlation was found between the patients’ communication self-efficacy and the number of QoL-topics discussed during consultations.

#### Satisfaction with communication

MCQ-C scores were higher in the intervention group at baseline in comparison with the control group (72 versus 63, *p* < 0.05). The higher scores remained at the consecutive time points. There were no changes in scores over time and no between-group difference in scores over time was seen.

#### Evaluation of the intervention

Ninety percent of patients reported that it would be useful to introduce the QoL-monitor as a standard instrument during treatment. The median time to complete the monitor was 10 min (IQR 7.5–15), patients did not perceive the monitor to be burdensome (median score 2) or difficult to complete (median score 1). Most patients believed the monitor was useful for the interaction with their HCP’s (median score 4) and suitable for documentation (median score 5).

## Discussion

In this study we found that introduction of routine assessment of quality of life during chemotherapy, with feedback to patients and healthcare professionals, can result in more health related QoL-topics being discussed and in a higher patients’ satisfaction with communication. More frequent discussions of psychosocial and breast cancer specific issues were observed in the intervention arm of our study. However, the increase in number of topics being discussed did not affect patients’ management. Between-group differences were not seen, either in secondary outcomes such as perceived quality of life, emotional distress or illness perceptions or self-efficacy. Patients felt that monitoring quality of use during the treatment period with chemotherapy resulted in a substantial benefit of their medical care.

Our findings are in agreement with previous reported studies, in which routine QoL assessments with feedback to patients and HCP’s were investigated in oncology patients [6, 7, 32]. An increase in discussion of QoL-topics and more frequent discussion of nonspecific chronic symptoms and improvement in patients’ wellbeing was in general observed after incorporating standardized QoL assessments [7]. No effect on patients’ medical management was observed in our study, which is consistent with earlier studies [7, 32]. In contrast with previous reports, the length of consultations was somewhat prolonged in the intervention group with an average of 2 min and 26 s. Possibly some QoL-topics were discussed more extensively when the QoL-monitor was used.

The increase in number of QoL-topics being discussed was limited and therefore one could argue whether this is clinically relevant. Especially when one considers that this is accompanied by somewhat longer consultation time. However, the higher scores on satisfaction with communication and the favorable evaluation of the monitor by patients, suggest that patients do benefit from the intervention. The relatively small difference of the discussed QoL-topics could be explained by the fact that all patients filled out questionnaires about QoL, illness perceptions and self-efficacy. This might encourage patients to discuss their issues with their HCPs. Besides, awareness of QoL-topics in participating HCPs might have resulted in more frequent discussion of QoL-topics in the standard care group as well. Before the start of the trial all HCP’s were informed in a plenary session about the aim of the study, how to interpret QoL scores and discussing results during the study visits. Although this awareness might give a contamination bias with a possible advantage for patients who received standard care as well, this is not necessarily an undesirable effect.

In this study we did not find a substantial improvement in self-efficacy after introducing the intervention. This could be explained by the high levels of self-efficacy in all participating patients at baseline, leaving little room for improvement. This so called ‘ceiling’ effect has been reported before in quality of life studies in cancer patients when questionnaires are used [7, 33, 34]. This might reflect a selection bias in this trial, as eloquent and assertive patients probably tended more likely to participate in a communication study. In addition, the study population consists of relatively young patients, treated in the Leiden University Medical Center or a teaching hospital nearby. Therefore, our results on self-efficacy may not reflect average scores in all patients with breast cancer in the Netherlands.

Our study has some limitations. In only 39% of patients all four study visits were recorded on tape and in 68% of patients sufficient audio-recordings were available to investigate the effect of the intervention. Audio-recordings were missed in most cases because the HCP’s forgot to start recording or because of technical failings. Loss to follow-up or patients who stopped chemotherapy was negligible. However, the amount of missing data did not differ between study arms. Therefore, we expect this had limited effect on our study results. We are not informed whether the HCPs actually reviewed the results from the QoL-monitor. However, two strategies were used to increase chances that the information was assessed: HCP’s received a notice that the patient had completed a new questionnaire that had been added to their electronic files and patients received a copy of their results and were asked to hand them to their HCP during the visit. Furthermore, the group of participating healthcare professionals had a heterogeneous background. Medical oncologists, medical oncologist in training and nurse practitioners were involved in this trial. Because of the relatively small sample size in this study the difference in HCP’s was not included as a covariate in the data analyses. Finally the results of this analysis of Dutch patients should be considered with caution, as the study was powered for the total group of Japanese and Dutch patients and not for this subgroup, neither for all the secondary outcome measures. A study with a larger sample size is needed to confirm these findings. The Japanese results and the combined findings of Dutch and Japanese patients are awaited.

Our study results suggest that communication between HCP’s and patient benefits from routine QoL assessments during breast cancer treatment with chemotherapy. Audio-recordings to investigate effects on patient-physician communication in our trial turned out to be an useful clinical research tool. Routinely assessment of QoL in patients with breast cancer being treated with systemic therapies should be standard of care and implemented in the guidelines. The QoL-monitor used in this trial is a modern and efficient tool that might be suitable for many patients and HCP’s. Further studies should be carried out to assess which patients benefit most from this intervention. We assume that introducing routine QoL assessments leads to a benefit for patients with other tumor types during their treatment as well. Furthermore, this intervention might not only be of importance for patients treated with chemotherapy, but also in patients treated with oral targeted therapies, immunotherapy or adjuvant hormonal therapy.

## Conclusion

The results of this study indicate that the use of a QoL-monitor during breast cancer treatment with chemotherapy, might lead to a more frequent discussion of health related quality of life topics, especially the psychosocial and breast cancer specific issues. Most patients appreciated the use of this QoL-monitor. Patients management and patients’ QoL was unaffected by the intervention.

## Electronic supplementary material

Below is the link to the electronic supplementary material.

Supplementary file1 (DOC 79 kb)

## Abbreviations

AC: Adriamycin and cyclophosphamide
AUC: Area under the curve
CNB: Care notebook
DT: Distress thermometer
EORTC-QLQ C30: European Organization for Research and Treatment of Cancer-Core Quality of Life Questionnaire
FEC: Fluorouracil, epirubicin and cyclophosphamide
HADS: Hospital Anxiety and Depression Scale
HCP: Healthcare providers
HRQL: Health related quality of life
MCQ-C: Medical Care Questionnaire-Communication
NCCN: National comprehensive cancer network
PEPPI: Perceived efficacy in patient-physician interactions
PRO: Patient-reported outcomes
PTC-Ptz: Paclitaxel, trastuzumab, carboplatin and pertuzumab
QoL: Quality of life
SD: Standard deviation
TAC: Docetaxel, adriamycin and cyclophosphamide
TC: Docetaxel and cyclophosphamide
