# Supplementary material for: Using a quality of life (QoL)-monitor: preliminary results of a randomized trial in Dutch patients with early breast cancer
Source: Qual Life Res. 2020 Jun 11;29(11):2961–75. doi: 10.1007/s11136-020-02549-8 (PMC7591431; doi:10.1007/s11136-020-02549-8)
Supplement: Supplementary file 1 — Supplementary file1 (DOC 79 kb) [file 11136_2020_2549_MOESM1_ESM.doc]

**Communication check list**

Patient no: Date:

Duration consultation (mm:ss): Duration physical exam (mm:ss):

In this check list we would like to ask you for each specific topic whether it has been discussed during the visit, who initiated the discussion of this topic, and which decisions or actions resulted from the discussion.

| **1** | | **2** | **3** |
| --- | --- | --- | --- |
| Please indicate for each of the following topics **whether is has been discussed** during today’s visit (Yes / No) | | If this topic has been discussed, please indicate **who** has initiated this discussion. | Which decisions or actions have resulted from the discussion of this topic? (for each question you answered with “YES”, please choose one or more numbers from the options list below)  0) no action / no decision  1) initiation of new medical treatment (e.g.  medication),  2) change in current chemotherapy plan,  3) referral to… (please indicate),  4) additional tests ordered (e.g. blood / MRI)  5) advice or counseling regarding self-care  6) explanation by doctor about the symptom  7) decided to wait and see  8) something else….(please indicate) |
| **1. Pain** | O Yes🡪  O No | O The doctor  O Me  O A relative or  friend | …………………………………………………  ………………………………………………… |
| **2. Fatigue** | O Yes🡪  O No | O The doctor  O Me  O A relative or  friend | …………………………………………………  ………………………………………………… |
| **3. Dyspnea** (shortness of breath) | O Yes🡪  O No | O The doctor  O Me  O A relative or  friend | …………………………………………………  ………………………………………………… |
| **4. Nausea or vomiting** | O Yes🡪  O No | O The doctor  O Me  O A relative or  friend | …………………………………………………  ………………………………………………… |
| **5. Sleep or sleep problems** | O Yes🡪  O No | O The doctor  O Me  O A relative or  friend | …………………………………………………  ………………………………………………… |
| **6. (Loss of) appetite** | O Yes🡪  O No | O The doctor  O Me  O A relative or  friend | …………………………………………………  ………………………………………………… |
| **7. Obstipation** | O Yes🡪  O No | O The doctor  O Me  O A relative or  friend | …………………………………………………  ………………………………………………… |
| **8. Diarrhea** | O Yes🡪  O No | O The doctor  O Me  O A relative or  friend | …………………………………………………  ………………………………………………… |
| **9.Side effects of chemotherapy**  (e.g. feeling ill, dry mouth, irritated eyes, differences in taste, hair loss, hot flushes) | O Yes🡪  O No | O The doctor  O Me  O A relative or  friend | …………………………………………………  ………………………………………………… |
| **10. Arm problems**  (e.g. pain, difficulty in moving or using the arm, swollen arm or hand) | O Yes🡪  O No | O The doctor  O Me  O A relative or  friend | …………………………………………………  ………………………………………………… |
| **11. Breast problems**  (e.g. pain, oversensitive breast, skin problems around the breast, swelling of breast area) | O Yes🡪  O No | O The doctor  O Me  O A relative or  friend | …………………………………………………  ………………………………………………… |
| **12. The consequences of illness and treatment for physical activities**  (e.g. lifting an object, walking, washing, dressing/undressing) | O Yes🡪  O No | O The doctor  O Me  O A relative or  friend | …………………………………………………  ………………………………………………… |
| **13. The consequences of illness and treatment for daily activities**  (e.g. household chores, work/employment) | O Yes🡪  O No | O The doctor  O Me  O A relative or  friend | …………………………………………………  ………………………………………………… |
| **14. The consequences of illness and**  **treatment for the relationship with others**  (e.g. your partner, children, family members,  friends) | O Yes🡪  O No | O The doctor  O Me  O A relative or  friend | …………………………………………………  ………………………………………………… |
| **15. The consequences of illness and**  **treatment for concentration and memory**  (e.g. reading, watching TV,  remembering things) | O Yes🡪  O No | O The doctor  O Me  O A relative or  friend | …………………………………………………  ………………………………………………… |
| **16.Your feelings and emotions**  (e.g. worries, fears, sadness, irritations,  anger) | O Yes🡪  O No | O The doctor  O Me  O A relative or  friend | …………………………………………………  ………………………………………………… |
| **17. The consequences of illness and treatment for intimacy and sexuality** | O Yes🡪  O No | O The doctor  O Me  O A relative or  friend | …………………………………………………  ………………………………………………… |
| **18. The consequences of illness and**  **treatment for your body image** (e.g.  satisfaction with bodily appearance,  feeling feminine, feeling attractive) | O Yes🡪  O N | O The doctor  O Me  O A relative or  friend | …………………………………………………  ………………………………………………… |
| **19. Possible worries about your health in the future** | O Yes🡪  O No | O The doctor  O Me  O A relative or  friend | …………………………………………………  ………………………………………………… |
| **20. Another topic (please write down):** | O Yes🡪  O No | O The doctor  O Me  O A relative or  friend | …………………………………………………  ………………………………………………… |
| **21. Another topic (please write down):** | O Yes🡪  O No | O The doctor  O Me  O A relative or  friend | …………………………………………………  ………………………………………………… |
| **22. Another topic (please write down):** | O Yes🡪  O No | O The doctor  O Me  O A relative or  friend | …………………………………………………  ………………………………………………… |
| **23. Another topic (please write down):** | O Yes🡪  O No | O The doctor  O Me  O A relative or  friend | …………………………………………………  ………………………………………………… |
| **24. Another topic (please write down):** | O Yes🡪  O No | O The doctor  O Me  O A relative or  friend | …………………………………………………  ………………………………………………… |
| **25. Another topic (please write down):** | O Yes🡪  O No | O The doctor  O Me  O A relative or  friend | …………………………………………………  ………………………………………………… |
